# Two face masks are better than one: congruency effects in face matching

**DOI:** 10.1186/s41235-022-00402-9

**Published:** 2022-06-08

**Authors:** Alejandro J. Estudillo, Hoo Keat Wong

**Affiliations:** 1 Department of Psychology, Bournemouth University, Poole, BH12 5BB UK; 2 University of Nottingham Malaysia, Semenyih, Malaysia

## Abstract

Although the positive effects of congruency between stimuli are well replicated in face memory paradigms, mixed findings have been found in face matching. Due to the current COVID-19 pandemic, face masks are now very common during daily life outdoor activities. Thus, the present study aims to further explore congruency effects in matching faces partially occluded by surgical masks. Observers performed a face matching task consisting of pairs of faces presented in full view (i.e., full-view condition), pairs of faces in which only one of the faces had a mask (i.e., one-mask condition), and pairs of faces in which both faces had a mask (i.e., two-mask condition). Although face masks disrupted performance in identity match and identity mismatch trials, in match trials, we found better performance in the two-mask condition compared to the one-mask condition. This finding highlights the importance of congruency between stimuli on face matching when telling faces together.

## Significance statement

COVID-19 has led to important changes in the way that humans communicate and interact with each other. To reduce contagion risk, face masks are now common during outdoor activities. However, as face masks conceal the bottom part of the face, they can have important consequences not only for social interactions but also in those scenarios where the identification of others is paramount, such as during ID verification, passport control and criminal investigations. One task that reproduces these scenarios under laboratory conditions is the face matching task, in which observers have to decide whether two simultaneously presented pictures depict the same (i.e., identity match) or two different people (i.e., identity mismatch). This study explores the effect of masks on face matching and whether congruency between both faces (i.e., both faces of the pair wear or do not wear a mask) improves performance in comparison with an incongruent condition (i.e., only one face of the pair wears a mask). Although face masks disrupted observers’ abilities to do the task, for identity match trials, we found better performance when both faces of the pair were wearing masks or were not wearing masks compared to the incongruent condition. This finding highlights the importance of congruency between stimuli on face matching when telling faces together.

## Introduction

In the face matching task, observers are simultaneously presented with two faces and have to decide whether these faces depict the same (i.e., identity matches) or two different identities (i.e., identity mismatches) (Bindemann, 2021; Bruce et al., 2001; Burton et al., 2010; Estudillo & Bindemann, 2014; Fysh & Bindemann, 2017; Johnston & Bindemann, 2013). At a theoretical level, the face matching task has contributed to our understanding of different face processing effects, including holistic processing in the perception of faces (Hole, 1994), the role of pictorial and identity codes during face identification (Menon et al., 2015), the cognitive locus of the other-race effect (Kokje et al., 2018; Megreya et al., 2011) and the effect of changing the viewpoint on face identification performance (Estudillo & Bindemann, 2014; Kramer & Reynolds, 2018), among others. This task is also considered the lab equivalent of the identification routines performed in security settings, such as ID verification, passport control, and criminal investigations (Bindemann, 2021).

Despite its apparent simplicity, matching two unfamiliar faces is challenging and error-prone (Alenezi & Bindemann, 2013; Fysh & Bindemann, 2017; Johnston & Edmonds, 2009; Johnston & Bindemann, 2013). Two non-exclusive sources of variation can explain these errors (Fysh & Bindemann, 2017). One of these sources is related to the observers’ actual skills to match faces (i.e., the so-called resource limit account). Indeed, research has shown that unfamiliar face matching skills present substantial individual differences across observers, with some individuals performing at chance levels while others performing at ceiling levels (Bruce et al., 2018; Burton et al., 2010; Estudillo & Bindemann, 2014; Estudillo et al., 2021; McCaffery et al., 2018). Thus, this account highlights the importance of using objective face identification tasks during personnel selection for those applied settings whereby the identification of others is demanded (Bobak et al., 2016; Estudillo, 2021;Estudillo & Wong, 2021; Fysh et al., 2020; Ramon et al., 2019; Robertson et al., 2016). In addition, according to the data limit account, a large variance of errors in face matching can be explained by the properties of the face stimuli (Estudillo & Bindemann, 2014; Fysh & Bindemann, 2017). Supporting this account, research has reported that image manipulations, such as pixelation (Bindemann et al., 2013), inversion (Megreya & Burton, 2006), feature masking (Carragher & Hancock, 2020; Estudillo et al., 2021; Noyes et al., 2021) and profile views (Estudillo & Bindemann, 2014; Kramer & Reynolds, 2018) impair unfamiliar face matching.

Behavioral and neuropsychological studies also suggest that the processes of telling faces together (i.e., identity match trials) and telling faces apart (i.e., identity mismatch trials) reflect different cognitive mechanisms (Bindemann & Burton, 2021). In fact, the performance in identity match and identity mismatch trials is not correlated (Megreya & Burton, 2007). Similarly, some experimental manipulations such as task duration (Alenezi & Bindemann, 2013; Alenezi et al., 2015), face images variability (Ritchie & Burton, 2017; Ritchie et al., 2021), image degradation (Bindemann et al., 2013) and feature-by-feature training (Megreya, 2018; Megreya & Bindemann, 2018; Towler et al., 2021) have different effects on identity match and identity mismatch trials. In addition, developmental prosopagnosics—people with lifelong face identification deficits (Duchaine & Nakayama, 2006)—tend to have specific problems in match but not in mismatch trials (Berger et al., 2022; Fisher et al., 2017; Mishra et al., 2021; White et al., 2017). Altogether, these results highlight the importance of considering the dissociable effects that different experimental manipulations might have on match and mismatch trials.

The outbreak of COVID-19 pandemic has led to important changes in the way that humans communicate and interact with each other. For instance, social distancing measures required citizens to maintain some physical distance with others. In addition, to reduce contagion risk, face masks are now common during outdoor activities. However, as face masks conceal the bottom part of the face (i.e., mouth and nose), they can have important consequences for social interactions and identification. Recent research has also explored the effects of face masks on face matching performance (Carragher & Hancock, 2020; Noyes et al., 2021). Despite large differences across individuals (Estudillo et al., 2021), face masks impair overall face matching performance (Carragher & Hancock, 2020; Noyes et al., 2021). Thus, as wearing face masks is becoming highly common in our daily life, it is important to identify the circumstances that maximize the matching accuracy of masked faces. Interestingly, some observers are able to match masked faces with remarkably high accuracy, pointing to the importance of personnel selection in security settings (Estudillo et al., 2021; Noyes et al., 2021). However, as aforementioned, a large variance of errors in face matching has its origin in the properties of the face stimuli (Estudillo & Bindemann, 2014; Fysh & Bindemann, 2017), suggesting that certain stimulus features might enhance face matching for masked faces.

One of these stimulus features could be contextual congruency. The positive effects of contextual congruency are well known in memory research (Chandler & Fisher, 1996). For example, in face recognition paradigms, it has been shown that faces encoded either in full view or wearing a headscarf are better recognized in a subsequent recognition stage when these faces are presented in the same condition in which they were learned, that is either in full view or with a headscarf (Toseeb et al., 2014). Similarly, using an eyewitness line-up paradigm, an advantage to identify faces with a ski-mask has been found when these faces were also encoded with a ski-mask, compared to when the face was studied in full view (Manley et al., 2019). These findings reveal that congruency between stimuli has a positive effect on face memory.

However, mixed findings have been reported regarding the effect of congruency on face matching. For example, some research has shown that participants were better at matching faces when both faces of the pairs were wearing glasses or were not wearing glasses, compared to a condition in which only one of the faces was wearing glasses (Kramer & Ritchie, 2016), and this effect seems to be driven by match trials (Graham & Ritchie, 2019). A more recent study found that hue congruency (i.e., both faces in color, in grayscale or mixed) does not affect face matching performance, but observers were more prone to make match responses in the incongruent condition compared to the congruent conditions (Bobak et al., 2019). More relevant to our purpose, a recent study comparing matching performance when only one or both faces of a pair had a face mask did not find any advantage of the two-mask condition compared to the one-mask condition (Carragher & Hancock, 2020).

At least three differences between these studies could explain the conflicting results. First, face masks, glasses and image hue differ in both the amount and type of facial information covered. That is, while image hue does not conceal any facial features and glasses only cover some information in the eye region, face masks conceal approximately 50% of the face, covering the mouth and nose. These differences are not irrelevant, as previous research has shown different roles of the eyes and mouth areas in face identification (Hills & Pake, 2013; Mckelvie, 1976; Peterson & Eckstein, 2012). In addition, while face masks qualitatively disrupt the way that faces are naturally processed (Freud et al., 2020), glasses and image hue do not seem to produce such a change in processing (Retter & Rossion, 2015; Righi et al., 2012). Thus, differences between stimuli might explain the conflicting results. Second, while Kramer and Ritchie (2016) and Graham and Ritchie (2019) used a within-participants design, Carragher and Hancock (2020) had different groups of participants performing each viewing condition, so it is also possible that potential differences between observers could hide any potential congruency effect in this latter study. Finally, Carragher and Hancock (2020) did not analyze the performance for match and mismatch trials separately. However, as previously mentioned, some research has found that these types of trials reflect different cognitive processes (Megreya & Burton, 2007), which could indeed explain why some researchers found congruency effects in match trials but not in mismatch trials (Graham & Ritchie, 2019).

The present study seeks to further investigate the effects of congruency and face masks on unfamiliar face matching. Observers performed an unfamiliar face matching task with three different viewing conditions: a full-view condition (i.e., both faces are presented in full view), a one-mask condition (i.e., only one face of the pair has a mask), and a two-mask condition (i.e., both faces of the pair have a mask). The aim of this study is twofold. First, we examine congruency effects on face matching, as previous studies have reported mixed findings (Carragher & Hancock, 2020; Graham & Ritchie, 2019; Kramer & Ritchie, 2016). If face matching is susceptible to congruency effects, we would expect to find better performance when both faces are presented with or without masks compared to the one-mask condition. Second, as previous research has found some dissociations between identity match and identity mismatch trials (Megreya & Burton, 2007), we explored whether the congruency and the mask effects are modulated by the types of identity trials.

## Method

### Participants

Participants were tested over the web, but the study could only be run using a PC or a laptop (no phones or tablets). Our initial sample consisted of 113 participants recruited using the platform Testable Minds (www.testable.org; Rezlescu et al., 2020). Ten participants were removed from further analysis due to performance below chance level and/or abnormally fast response times (< 400 ms). Thus, our final sample consisted of 103 participants (34 females) with a mean age of 29 years (SD = 8.69). Retrospective power analysis run with the software MorePower (Campbell & Thompson, 2012) revealed that with 103 participants and a power of 0.80 we would detect a small effect of 0.04, which is substantially smaller than those congruency effects previously reported in face matching (Kramer & Ritchie, 2016). Participants gave their consent to participate in this study and received 3 USD as compensation for their time. This study was approved by the research ethics committee of Bournemouth University.

### Stimuli

**Fig. 1:**
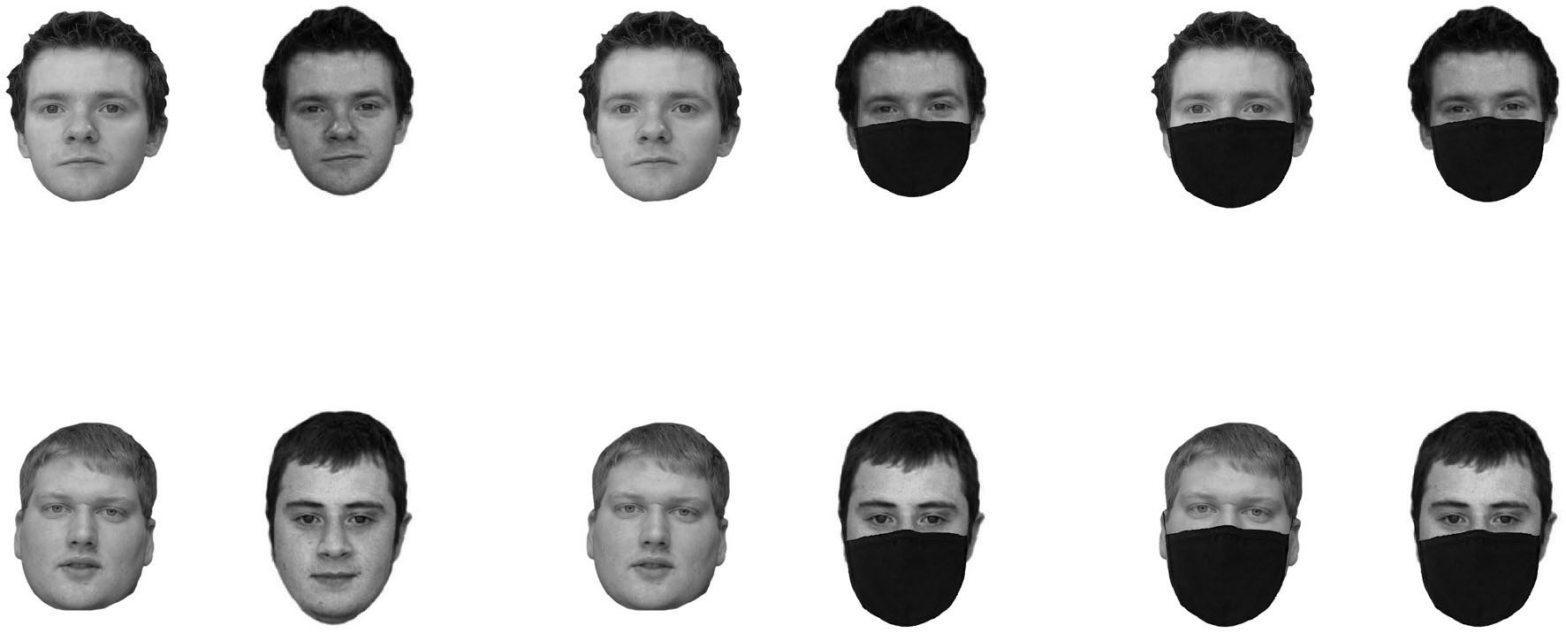
Example stimuli depicting identity matches (top row) and identity mismatches (bottom row) in the full view (left column), one-masked face (middle column), and two-mask conditions (right column)

One hundred and twenty pairs of female and male Caucasian faces from the Glasgow Unfamiliar Face Database (Burton et al., 2010) were used in this study. One face photograph in each pair was taken with a high-quality digital camera, while the other was a still frame from high-quality video. All faces were shown in greyscale on a white background, measuring 700 × 500 pixels at a resolution of 72 ppi. There was a distance of approximately 250 pixels between both faces. There were three viewing conditions, with 40 pairs in each of them. In the *full-view condition*, both faces were presented without a face mask. In the *one-mask condition*, one face of the pair was presented in full view, while the other face was presented with a face mask. In this condition, the position of the face mask (left face vs. right face) was counterbalanced across trials. Finally, in the *two-mask condition*, both faces were presented with a face mask. The allocation of the face pairs to these conditions was randomized across participants. Half of the trials depicted two pictures from the same identity (i.e., identity match trials), while the other half depicted two pictures from two different people (i.e., identity mismatch trials). Following previous studies (i.e., Estudillo et al., in press: Carragher & Hancock, 2020), we used Photoshop to fit the face masks to the face stimuli. However, to ensure that the masks cover the same features as a real mask would, this process was done individually for each face. Stimuli examples are presented in Fig. 1.

### Procedure

On each trial, observers were firstly shown a central fixation cross for 1000 ms, which was followed by two face images presented side by side. Observers had to decide whether the two pictures depicted the same or two different identities by pressing one of two buttons. The faces remained onscreen until a response was made. Trials were randomized across participants. A demo of this task can be found at https://www.testable.org/experiment/4045/339472/start

## Results

**Fig. 2:**
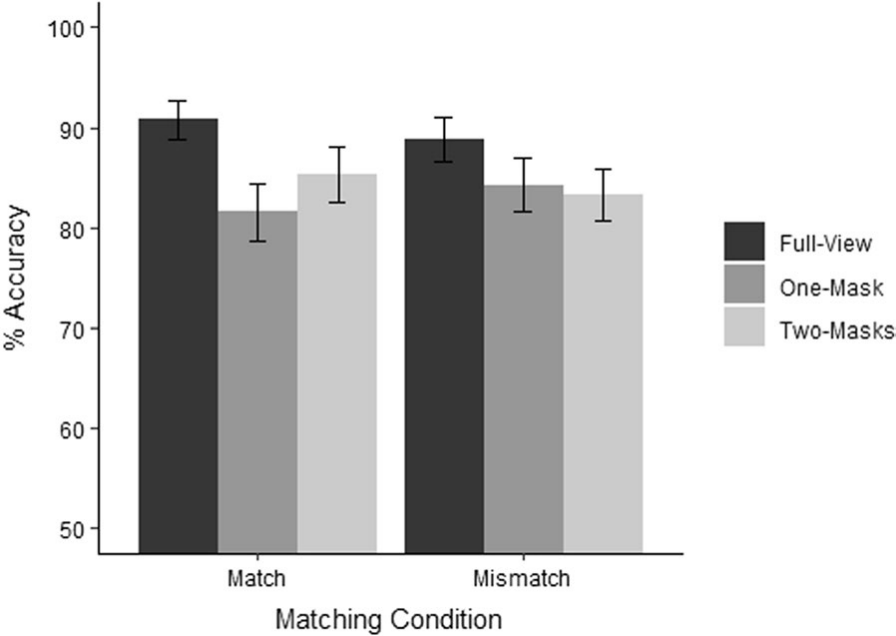
Mean percentage accuracy across viewing and identity conditions

The percentage of correct responses across viewing conditions is presented in Fig. 2. A 3 (viewing condition: full-view vs. one-mask vs. two-masks) × 2 (identity condition: identity match vs. identity mismatch) repeated -measures ANOVA revealed that the main effect of identity condition was not statistically significant, *F*(1, 102) = 0.05, *p* = *0.80.* The main effect of viewing condition reached statistical significance *F*(2, 204) = 42.68, *p* < *0.001,* *η*^2^_p_ = 0.29. This main effect was qualified by a significant interaction with viewing condition, *F*(2, 204) = 5.43, *p* < *0.01,* *η*^2^_p_ = 0.05*.* Simple main effect analysis revealed that the effect of viewing condition was significant for match trials, *F*(2, 204) = 30.48, *p* < *0.001,* *η*^2^_*p*_ = 0.23*.* Post hoc t-test (Holm–Bonferroni-corrected) showed better performance in the full-view condition (*M* = 90.82, SD = 9.95) compared to both the one-mask condition (*M* = 81.55, SD = 14.63) and the two-mask condition (*M* = 85.29, SD = 13.93), both *ts*(102) ≥ 4.63, *ps* < 0.001, *ds* ≥ 0.45. Performance was also better in the two-mask condition compared to the one-mask condition, *t*(102) = 3.12, *p* < 0.01, *d* = 0.30. The effect of viewing condition was also significant for mismatch trials, *F*(2, 204) = 14.41, *p* < *0.001,* *η*^2^_*p*_ = 0.12. Post hoc analysis revealed better performance in the full-view condition (*M* = 88.78, SD = 11.59) compared to both the one-mask condition (*M* = 84.27, SD = 13.43) and the two-mask condition (*M* = 83.35, SD = 13.16), both *ts*(102) ≥ 4.16, *ps* < 0.001, *ds* ≥ 0.41. However, the performance was similar for the one-mask and the two-mask conditions, *t*(102) = 0.85, *p* = 0.39.

Signal detection theory measures were also analyzed. Specifically, correctly identified match trials (i.e., hits) and incorrectly identified mismatch trials (i.e., false alarms) were used to calculate d-prime and C (Stanislaw & Todorov, 1999). D-prime is a measure of discriminability between match and mismatch trials, and higher values indicate better discriminability. D-prime for extreme values (i.e., perfect accuracy) were corrected using Hautus’ recommendations (Hautus, 1995). C is a measure of response bias. Positive values suggest that observers are more prone to making mismatch responses (i.e., conservative bias), while negative values indicate that observers are more prone to making match responses (i.e., liberal bias).

D-prime and C values across viewing conditions are presented in Table 1. A one-way ANOVA on d-prime scores revealed a main effect of viewing condition, *F*(2, 204) = 43.47, *p* < *0.001,* *η*^*2*^_*p*_ = 0.29*.* Post hoc analysis revealed better discriminability in the full-view condition compared to both the one-mask and the two-mask conditions, both *ts*(102) ≥ 7.27, *ps* < 0.001, *ds* ≥ 0.72. However, performance was similar in the one-mask and the two-mask conditions, *t*(102) = 1.41, *p* = 0.16. The analogous analysis on C revealed a main effect of viewing condition, *F*(2, 204) = 5.83, *p* < *0.01,* *η*^2^_*p*_ = 0.05*.* Post hoc analysis showed a more conservative bias in the one-mask condition compared to both the full-view and the two-mask conditions, both *ts*(102) ≥ 2.73, *ps* < 0.03, *ds* ≥ 0.26. Response bias was similar in the full-view and the two-mask conditions, *t*(102) = 0.39, *p* = 0.69.

**Table 1:** Mean *d*-prime and *C* (standard deviations in brackets) in each viewing condition

	*D*-prime	*C*
Full-view	2.64 (.69)	− 0.04 (0.39)
One-mask	2.04 (.65)	0.06 (0.45)
Two-mask	2.14 (.74)	− 0.05 (0.39)

## Discussion

This study aimed to investigate the effects of face masks and congruency on unfamiliar face matching and whether these effects were modulated by the type of identity trial (i.e., identity match vs. identity mismatch). To achieve this, our observers performed a face matching task consisting of pairs of faces presented in full-view, pairs of faces in which only one of the faces had a mask, and pairs of faces in which both faces had a mask. Our results reveal three interesting patterns. First, we found that face masks had a detrimental effect on face matching, and this effect was equivalent for identity match and identity mismatch trials. Second, in identity match trials, we found better performance in the full-view and the two-mask conditions compared to the one-mask condition. In contrast, in identity mismatch trials, although participants’ accuracy was better in the full-view condition compared to both one-mask and two-mask conditions, performance was similar in these two latter conditions. Third, observers were more prone to making mismatch responses in the one-mask condition compared to both the full-view and the two-mask conditions.

Our results are in agreement with recent research showing that, although with large individual differences (Estudillo et al., 2021), face masks generally disrupt overall face matching performance (Carragher & Hancock, 2020; Noyes et al., 2021). More interestingly, our results add to the existing literature that this impairment is observed across both identity match and identity mismatch trials. In other words, face masks impair both the ability to tell faces together and the ability to tell faces apart. This finding is remarkable as it contrasts with other manipulations, such as task duration (Alenezi & Bindemann, 2013; Alenezi et al., 2015), image variation (Ritchie & Burton, 2017; Ritchie et al., 2021), image degradation (Bindemann et al., 2013) and feature-by-feature training (Megreya, 2018; Megreya & Bindemann, 2018; Towler et al., 2021), which exclusively affect performance on either identity match or identity mismatch trials. As it has been previously suggested that these two types of trials reflect partially different cognitive operations (Megreya & Burton, 2006), our findings suggest that face masks would disrupt processes that are common to both identity match and identity mismatch trials. One possibility would be that face masks impair holistic processing, which is considered the hallmark of face perception (Estudillo, 2012; Rossion, 2013; Wong et al., 2021). This is supported by recent research that has shown that face masks have stronger effects on upright compared to inverted faces (Freud et al., 2020).

Although previous research has shown congruency effects in unfamiliar face matching (Bobak et al., 2019; Graham & Ritchie, 2019; Kramer & Ritchie, 2016), this congruency effect has not been recently replicated with face masks (Carragher & Hancock, 2020). In the current study, we have reported better performance in the congruent conditions (i.e., full-view and two-mask conditions) compared to the incongruent condition (i.e., one-mask condition). However, these congruency effects were only evident for match but not for mismatch trials (for similar results with glasses, see Graham & Ritchie, 2019). This congruency effect is remarkable as it suggests that adding extra facial information to one of the faces of the pair while keeping this information concealed in the other face of the pair is not only irrelevant to solve the task, but it also impairs matching performance.

Importantly, it seems that the congruency effects produced by masks are different to those reported with glasses (Kramer & Ritchie, 2016) or with hue manipulations on faces (Bobak et al., 2019). Two different patterns of results support this suggestion. First, although we found differences between the congruent conditions (i.e., better performance in the full-view compared to the two-mask condition), Kramer and Ritchie (2016) showed similar performance in both congruent conditions (i.e., full-view and both faces with glasses). Second, while Kramer and Ritchie (2016) and Bobak and colleagues (2019) reported that incongruency between faces increased the number of match responses, we found the opposite pattern. Likely, these differences can be explained in terms of stimulus differences between these studies. In fact, while face masks cover approximately half of the face, little facial information is lost as a consequence of wearing glasses or hue changes in the image.

Regardless of these differences, our results in conjunction with others (Bobak et al., 2019; Graham & Ritchie, 2019; Kramer & Ritchie, 2016) not only highlight the importance of contextual congruency to improve face matching performance, but also suggest that different types of contextual congruency manipulations that might lead to similar behavioral outcomes (i.e., changes in face identification performance) are potentially driven by different cognitive mechanisms. In the case of the current study, we tentatively suggest that the observed benefits of two masks compared to one mask in match trials are related to the interference from irrelevant facial features in the one-mask condition. Previous research using the part-whole paradigm (Tanaka & Farah, 1993; Tanaka & Simonyi, 2016; Wong et al., 2021) has shown that irrelevant facial features are so difficult to ignore, that they negatively affect the identification of target facial features (Leder & Carbon, 2005). In our study, when only one of the faces of the pair is wearing a face mask, observers can only base their decision on the top part of the face. However, as the bottom part of the full-view face is available, this part would interfere with the identification of the top part of the face by increasing the dissimilarity between both top parts. This would lead observers to think that the faces belong to different identities. In mismatch trials, observers have to reject two top parts as being the same, but in the one-mask condition, the bottom part of the full-view face does not decrease the dissimilarity between both top parts.

The reported congruency effects with face masks have important consequences for applied scenarios. Given that face masks are highly common nowadays, in ID verification settings, such as during passport control or the identification of a perpetrator, it might be necessary to match the identity of a full-view face with a face wearing a mask (e.g., when a suspect wearing a mask is caught by a CCTV camera). However, our results suggest that this scenario should be treated with caution as it increases the probability of a misidentification. Thus, asking a suspect to wear a mask—if the suspect is present during, for example, an identification parade—or even artificially superimposing a mask on a different picture (e.g., passport picture)—if the suspect is absent—could decrease the probability of a misidentification. Concerning this second option, it must be acknowledged that although superimposing a mask on face pictures has similar effects to when the face was actually wearing a mask, these effects seem to be stronger in the former case (Carragher & Hancock, 2020; Estudillo et al., 2021; Noyes et al., 2021). Possibly, these differences are due to the fact that shape information (e.g., the shape of the external contour) is more easily retained in natural pictures of people wearing masks.

Despite the relevance of the present findings, one important limitation of the present study must be noted. Previous reports studying the effect of face masks (Noyes et al., 2021) and congruency (Graham & Ritchie, 2019; Kramer & Ritchie, 2016) on unfamiliar face matching used ambient face stimuli. In contrast, in our study, we have used face stimuli from the Glasgow Unfamiliar Face Database (Burton et al., 2010), which are high-quality images that present low within-individual variability. Therefore, it could be argued that our results could be partially explained by the use of constrained face stimuli. However, this explanation is unlikely as other studies have reported similar mask effects using the Glasgow Unfamiliar Face database (Carragher & Hancock, 2020; Estudillo et al., 2021).

In conclusion, while our results show that face masks disrupt face matching, performance for match trials was better when both faces of the pair were wearing a face compared to when only one of the faces was wearing a face mask. This finding demonstrates that congruency within face pair improves face matching accuracy.

## Data Availability

Data are available upon request.
